# Phospholipase A_2_ in chamber angle of normal eyes and patients with primary open angle glaucoma and exfoliation glaucoma

**Published:** 2007-03-26

**Authors:** Seppo Rönkkö, Petri Rekonen, Kai Kaarniranta, Tuomo Puustjärvi, Markku Teräsvirta, Hannu Uusitalo

**Affiliations:** 1 Department of Ophthalmology, University of Kuopio, Kuopio, Finland; 2 Department of Ophthalmology, Kuopio University Hospital, Kuopio, Finland

## Abstract

**Purpose:**

Phospholipase A_2_ (PLA_2_) is a growing family of lipolytic enzymes that play a key role in various biological processes including general lipid metabolism, membrane homeostasis, and in diseases such as atherosclerosis, arthritis, and acute pancreatitis. Oxidative stress as well as inflammation may be associated with glaucoma pathogenesis. Therefore, our aim was to examine the expression of group IIA secretory PLA_2_ (sPLA_2_-IIA), group V secretory PLA_2_ (sPLA_2_-V), calcium-independent PLA_2_ (iPLA_2_), and cytosolic PLA_2_ (cPLA_2_) type in the trabecular meshwork (TM) and the canal of Schlemm in normal eyes and in juxtacanalicular tissue samples from patients with primary open angle glaucoma (POAG) or exfoliation glaucoma (ExG).

**Methods:**

TM tissues were isolated from healthy donor eyes for corneal transplantation. Specimens of inner wall of the Schlemm's canal and the juxtacanalicular tissue were collected during deep sclerectomy from the eyes of patients who had POAG or ExG. Antibodies against PLA_2_s (sPLA_2_-IIA, sPLA_2_-V, iPLA_2_, and cPLA_2_) and a standard immunohistochemical procedure were used for the analysis. Quantification of immunoreactions was provided using a Photoshop-based image analysis. Double-staining immunofluorescence of macrophages and sPLA_2_-IIA was performed by using confocal microscopy.

**Results:**

sPLA_2_-IIA was not present in normal TM. In contrast, sPLA_2_-IIA levels were significantly higher in glaucoma patients than in controls. Furthermore, sPLA_2_-IIA expression was much higher in POAG when compared to ExG. iPLA_2_ was found to predominate in normal human TM, and it demonstrated strong labeling in the uveal and corneoscleral meshwork. The staining of juxtacanalicular meshwork was only moderate in density. In contrast, expression of the enzyme was significantly decreased in glaucoma patients, especially in ExG, when compared to normal controls or to POAG. In addition, strong regional differences were detected in sPLA_2_-IIA and iPLA_2_ levels in POAG, whereas immunostaining of these enzymes was much lower and rather uniform throughout ExG sample. In POAG, sPLA_2_-IIA staining was restricted to certain parts of the trabecular samples where sPLA_2_-IIA positive macrophages were also present. Immunostaining of sPLA_2_-V or cPLA_2_ was low, and no significant changes were found in levels of these enzymes between normal and glaucomatous samples.

**Conclusions:**

sPLA_2_-IIA, an oxidative stress marker in atherosclerosis, is overexpressed especially in POAG. This result supports the hypothesis that oxidative stress may play a significant role in the pathogenesis of POAG. In ExG, a dramatic decrease in the expression level of iPLA_2_, a housekeeping enzyme in phospholipid remodeling, may indicate imbalance in phospholipid turnover and also inhibition of normal physiological functions in the TM. These findings may contribute to understanding the pathogenesis of POAG and ExG and may be important for the development of novel therapeutic strategies to different glaucomas.

## Introduction

The term glaucoma is used to describe a heterogeneous group of diseases that have in common a characteristic optic cup neuropathy with loss of visual field defects [1]. Elevated intraocular pressure (IOP) is a strong risk factor for open-angle glaucoma, but some patients with glaucoma have normal IOP and many patients with elevated IOP do not have glaucoma [2,3]. In Finland and other Nordic countries, the most common types of glaucoma are primary open-angle glaucoma (POAG) and exfoliation glaucoma (ExG) [4-6]. Usually ExG is more aggressive; it reacts worse to medical treatment, and optic nerve damage and visual field loss take place earlier than in POAG [7-12]. Elevated IOP in ExG may be attributed to accumulation of the exfoliation material or pigment particles in the angle chamber [13-15].

PLA_2_ (EC 3.1.1.4) belongs to a superfamily of enzymes that catalyzes the hydrolysis of the sn-2 ester bond in phospholipids. The hydrolysis products are free fatty acids and lysophospholipids [16,17]. Different PLA_2_ isoenzymes have been found and classified into several groups (from I to XIV) based on their structures, subcellular distributions, cellular functions, and enzymatic characteristics [18,19]. In a simplified classification system, PLA_2_s can be divided into four major groups: secretory PLA_2_ (sPLA_2_), Ca^2+^-independent PLA_2_ (iPLA_2_), cytosolic PLA_2_ (cPLA_2_), and a class of PLA_2_ called platelet-activating factor (PAF) acetylhydrolase (PAF-AH) [20-22]. sPLA_2_ is optimally active at millimolar Ca^2+^ concentration and cPLA_2_ requires micromolar amounts of Ca^2+^, whereas iPLA_2_ does not need Ca^2+^ for activity [23].

PLA_2_s play a key role in various biological processes. sPLA_2_ has been implicated in the regulation of a wide array of cellular functions, such as arachidonic acid (AA) metabolism, phospholipids digestion, extracellular matrix (ECM) remodeling, regulation of proliferation and cell contraction, endothelial cell migration, antimicrobial defense, and regulation of acrosome reaction of spermatozoa [23-33]. Elevated levels of sPLA_2_ have been detected in several diseases including atherosclerosis, inflammatory diseases, arthritis, acute pancreatitis, and neurodegeneration [34-39].

cPLA_2_ is the only PLA_2_ that shows significant selectivity toward AA at the sn-2 position of the phospholipid molecule [40]. Therefore, it plays an important role in mediating important cellular processes including eicosanoid biosynthesis [41].

iPLA_2_ is generally regarded as a housekeeping enzyme as it remodels and maintains membrane phospholipids [42]. Recent studies have suggested the enzyme has other roles. iPLA_2_ has a proliferative effect and a functional role in cellular signaling cascades, vascular smooth muscle contraction, artery relaxation, and in apoptotic processes [43-48]. Recently, it was reported that iPLA_2_ is required for activation of store-operated Ca^2+^ channels to initiate Ca^2+^ influx [49].

In general, mammalian cells contain more than one PLA_2_ [17] thus there is considerable interest in determining the role of each PLA_2_. To our knowledge, although much research has been done to characterize, purify, and clone various forms of PLA_2_ from diverse sources, virtually nothing has been presented about the existence of PLA_2_ in the human anterior chamber angle. The anterior segment of the eye is filled with aqueous humor. A major component of the anterior chamber angle is the trabecular meshwork (TM) and the canal of Schlemm. The TM regulates the outflow facility of the aqueous humor and is also responsible for IOP control [50]. Recent notions that oxidative stress may play a role in glaucomatous TM cells have brought new insights to probable pathophysiologic mechanisms behind glaucoma [51]. Interestingly, PLA_2_s are important mediators of oxidative damage in cells [20,39]. To fill this gap, the goal of the present study was to immunohistochemically analyze the expression of PLA_2_ in normal human chamber angle and the inner wall of Schlemm's canal and the juxtacanalicular tissue of patients with POAG or ExG. In this work we used antibodies against four distinct PLA_2_s, including group IIA secretory PLA_2_ (sPLA_2_-IIA), group V secretory PLA2 (sPLA_2_-V), iPLA_2_, and cPLA_2_.

## Methods

### Materials

Monoclonal antibodies against sPLA_2_-IIA were purchased from Upstate (Lake Placid, NY). Monoclonal antibodies against sPLA2-V and cPLA2 (sc-4-4B-3C, lot number E1704) and rabbit polyclonal antibodies against iPLA_2_ were purchased from Santa Cruz Biotechnology, Inc (Santa Cruz, CA). Monoclonal antibodies against CD68 (Lab Vision Corporation) were used for immunohistochemical staining of macrophages. For the double labeling experiments goat antimouse IgG conjugated to Alexa Fluor 488 or Alexa Fluor 594 secondary antibodies (Molecular probes, Eugene OR) were used. For Western blot experiments, horseradish peroxidase-linked antimouse (GE Healthcare, Buckinghamshire, England) or antirabbit (GE Healthcare) secondary antibodies were used.

### Samples

Normal human TM tissue samples were obtained from healthy eyes donated for corneal transplantation (n=8). Tissue samples were also obtained from patients with POAG (n=6) and patients with ExG (n=6), who were undergoing elective deep sclerectomy. During the operation, the external wall of Schlemm's canal was opened, and the tissue specimens were taken from the inner wall of Schlemm's canal. The juxtacanalicular meshwork and corneoscleral trabecular layers were taken by direct visual control during the surgery. Tissue specimens were frozen in -70 °C until used, or they were fixed in formalin and embedded in paraffin. Paraffin sections (5 μm) and cryostat (Leica CM3050S, Leica Microsystems, Nussloch, Germany) sections (5 μm) were placed on Super Frost®Plus microscope slides (Menzel GmbH & Co KG, Germany). The procedure for obtaining the tissues was within the tenets of the Declaration of Helsinki.

### Clinical findings in primary open angle glaucoma and exfoliation glaucoma patients

Prior to the surgery, clinical data was collected on each patient, including age, gender, use of prostaglandin analogs, number of argon laser trabeculoplasty and other ocular surgical interventions, type and duration of glaucoma, IOP, and visual acuity. Glaucoma classification was based on careful clinical eye examination. All patients underwent slit lamp examination on the day before surgery. All IOPs in the POAG or ExG group exceeded 20 mmHg at the time of surgery. Visual acuity varied from 0.3 to 1.0.

### Homogenization and western blot analysis of control samples

Low molecular weight standards were obtained from Amersham Biosciences. Normal human TM tissue samples were homogenized on ice in T-PER Tissue Protein Extraction Reagent with protease inhibitor coctail (Pierce, Rockford, IL). Proteins (10 μg) were separated by SDS-PAGE [52], and after the run, the gels were subjected to western blot. Briefly, the samples were transferred (voltage: 12 V; current: 100 mA) to Hypond ECL (nitrocellulose) membranes (Amersham Biosciences) for 1 h using a semidry blotter (Transblot system, Bio-Rad, Hercules, CA). Transfer buffer was 25 mM Tris containing 192 mM glycine and 20% methanol. The membranes were blocked with 3% milk powder in phosphate-buffered saline (PBS) with 0.3% Tween for 1 h at 25 °C. After blocking the membranes were incubated overnight at 4 °C with antibodies directed against sPLA_2_-IIA, sPLA_2_-V, iPLA_2_, or cPLA_2_ (each with 1:500 dilution in blocking solution). Membranes were washed in PBS with 0.3% Tween three times for 10 min each. Membranes were probed with the appropriate secondary antibody (antimouse IgG used at 1:50,000 dilution in blocking solution or antirabbit IgG used at 1:20,000 dilution in blocking solution) linked to horseradish peroxidase for 2 h. Membranes were then washed in PBS with 0.3% Tween three times for 10 min each. Proteins were visualized with Immobilon Western Chemiluminescent HPR substrate (Millipore, Billerica, MA) and exposed to Fuji RX film (Fuji, Japan). Purified recombinant human sPLA_2_-IIA (BioVendor GmbH, Heidelberg, Germany) and sPLA_2_-V (BioVendor, GmbH) were used as positive controls.

### Immunohistochemistry

Cryosections were fixed in ice-cold acetone for 7 min, air-dried, then rinsed twice with tris-buffered saline (TBS). Paraffin sections were dewaxed in xylene and dehydrated in graded ethanols according to standard procedures. Immunostaining was carried out with HistostainTM-Plus Mouse Primary Bulk kit (Zymed Laboratories, South San Francisco, CA) or Histostain^TM^-Plus Broad Spectrum Bulk kit (Zymed Laboratories) and with DAB substrate kit (Zymed Laboratories) following guidelines described in reference [53]. The antibodies for demonstrating sPLA_2_-V, iPLA_2_, and cPLA_2_ were all used at a dilution of 1:100, and a dilution of 1:400 was used for sPLA_2_-IIA. The tissue sections were examined and digitally captured using a Nikon Eclipse TE300 inverted microscope (Nikon, Tokyo, Japan) equipped with Nikon E995 digital camera (Nikon), and the images were processed with Adobe Photoshop (version 5.5) software.

### Confocal laser scanning microscopy

Double-immunofluorescence was performed for colocalization studies of sPLA_2_ and macrophages using the method described in Kroeber et al. [54]. Primary antibodies used were anti-sPLA_2_-IIA (1:400 dilution) and anti-CD68 (1:10 dilution). sPLA_2_-IIA was detected with antimouse IgG Alexa Fluor 488 (1:200 dilution), and macrophages were detected with Alexa Fluor 594 (1:5 dilution; red fluorescence). In addition, nuclei were stained with a 1 mM solution of far red nucleic acid dye (SYTO 62; Molecular Probes).

For colocalization studies cryosections were fixed in ice-cold acetone for 7 min, air-dried, rinsed twice with TBS and blocked with blocking solution. Colocalization of sPLA_2_-IIA and macrophages was observed by merged images with UltraVIEW confocal imaging systems (PerkinElmer Life Sciences, Shelton, CT) following guidelines established in reference [55]. Sections were mounted on Vectashield mounting medium (Vector, Burtingame, CA). To verify an absence of cross-reaction between antibodies, we omitted each primary or secondary antibody from the incubation. All control experiments confirmed that there was no cross-reactivity between the antibodies.

### Quantification of immunohistochemical staining

The amount of antibody staining was quantified by using Photoshop-based image analysis [53]. All samples were analyzed in triplicate. The final immunostaining intensity (AU) was determined by subtracting the intensity of the negative control.

### Statistical analysis

Differences between experimental groups were determined using the Mann-Whitney Rank Sum test (SigmaStat statistical software, SPSS Inc, Chicago, IL). A p less than or equal to 0.05 was considered statistically significant.

## Results

### Distribution of sPLA_2_-IIA, sPLA_2_-V, iPLA_2_, and cPLA_2_ in normal human trabecular meshwork

**Figure 1:**
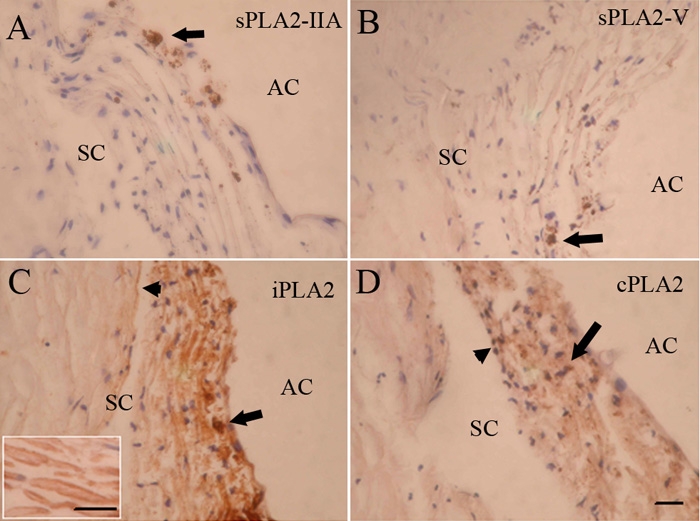
Immunohistochemical localization of PLA_2_s in normal human trabecular meshwork. Immunostaining for sPLA_2_-IIA (**A**) or sPLA_2_-V (**B**) was negative in the trabecular meshwork. Intense staining for sPLA_2_-IIA or sPLA_2_-V was evident in a few inflammatory-like cells (arrow). iPLA_2_ immunolabeling was strong (**C**). Labeling of the uveal and corneoscleral meshwork was stronger compared to the staining of the juxtacanalicular meshwork. Low positive staining was seen in the apical parts of the cells lining Schlemm's canal (arrowhead) as well as in nearby extracellular regions. Positive staining was also seen in a few macrophages (arrow). Inset: A portion of trabecular meshwork lamellae at higher magnification. Uveal trabecular meshwork cells covering the lamellae were more intensely labeled compared to connective tissue core. cPLA_2_ was weakly positive (**D**) and staining was slightly higher in uveal and corneoscleral meshwork compared to juxtacanalicular meshwork. The cells lining Schlemm's canal showed weak staining. Positive staining was seen in a few macrophages (arrow). AC, anterior chamber; SC, Schlemm's canal. The scale bar is equal to 50 μm.

We immunostained tissue sections to assess the immunohistochemical localization of sPLA_2_-IIA, PLA_2_-V, iPLA_2_, and cPLA_2_ in human TM. sPLA_2_-IIA (Figure 1A) or sPLA_2_-V (Figure 1B) immunohistochemical labeling was observed in few macrophages. In contrast, iPLA_2_ demonstrated strong labeling of the uveal and corneoscleral meshwork, and the staining of juxtacanalicular meshwork was only moderate in density. Uveal TM cells covering the lamellae were more intensely labeled compared to the connective tissue core of the lamellae, and, similarly, the luminal parts of the cells lining Schlemm's canal were noticeably stained with iPLA_2_. Staining intensity of cPLA_2_ was considerably weaker when compared to iPLA_2_. Immunohistochemical staining showed that cPLA_2_ expression was slightly higher in uveal and corneoscleral meshwork compared to juxtacanalicular meshwork (Figure 1D). The cells lining Schlemm's canal were also faintly stained with cPLA_2_. Moreover, intense staining of iPLA_2_ (Figure 1C) or cPLA_2_ (Figure 1D) was also seen in few macrophages. A similar pattern of PLA2s was found in paraffin-embedded and frozen sections of normal human tissue.

### Localization of sPLA_2_-IIA, sPLA_2_-V, iPLA_2_, and cPLA_2_ in trabecular meshwork of primary open angle glaucoma and exfoliation glaucoma patients

**Figure 2:**
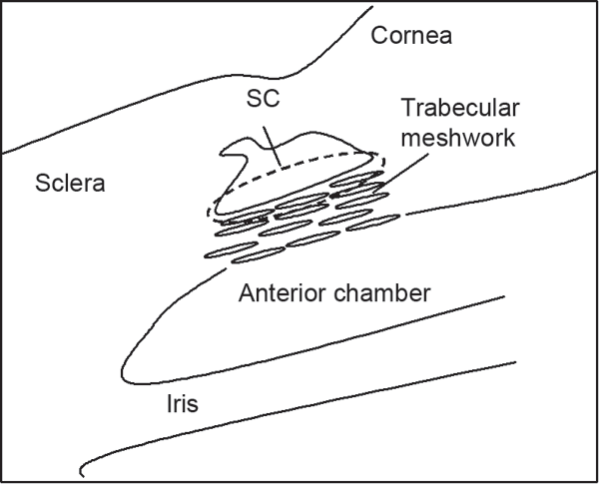
Surgical site. Shown in this schematic line drawing of the chamber angle is the location of the surgical site (dashed line). SC, Schlemm's canal

**Figure 3:**
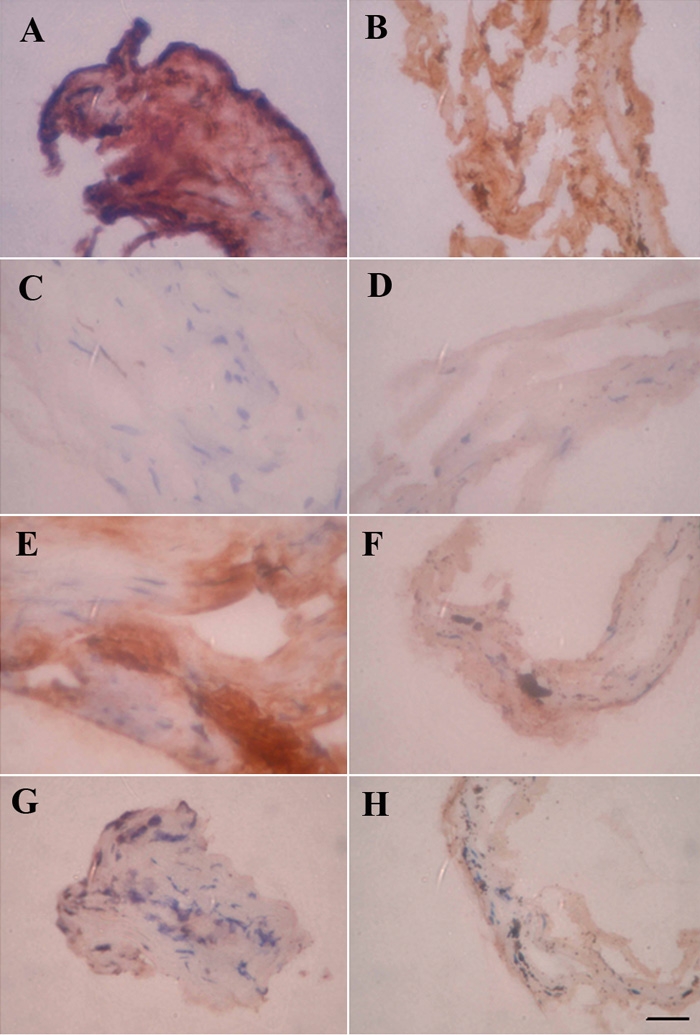
Immunostaining for PLA_2_s in POAG and ExG. Immunoreaction for sPLA_2_-IIA is heavier in POAG (**A**) compared to ExG (**B**). In trabecular meshwork tissue, immunoreactivity was found near the stained macrophages. PLA_2_-V staining was weak in POAG (**C**) and ExG (**D**). In POAG (**E**) trabecular meshwork staining for iPLA_2_ is near positively stained macrophages. In ExG samples (**F**) iPLA_2_ staining is in macrophages. Cellular staining of cPLA_2_ was low in POAG (**G**) and ExG (**H**) samples. The scale bar was equal to 50 μm.

We examined the locations of sPLA_2_-IIA, sPLA_2_-V, iPLA_2_, and cPLA_2_ in TM samples from eyes with glaucoma. POAG and ExG specimens were collected during deep sclerectomy by removing the inner wall of Schlemm's canal and adjacent juctacanalicular tissue and the TM but leaving the inner meshwork intact (Figure 2). In general, we found that immunoreaction of sPLA_2_-IIA was much heavier in POAG samples than that in ExG samples (Figure 3A,B). In POAG eyes, heavy immunoreactivity was seen in trabecular tissue and around the macrophages, which stained positive for sPLA_2_-IIA (Figure 3A). In ExG samples, strong positive PLA_2_-IIA staining was detectable mostly in few macrophage-like cells, and components of extracellular matrix were not so intensively stained compared to POAG samples (Figure 3B).

No apparent differences were seen in expression levels or localization of sPLA_2_-V between POAG and ExG samples (Figure 3C,D). Staining for sPLA_2_-V was weakly positive. The expression pattern of iPLA_2_ was different in POAG and ExG tissues. In POAG eyes, iPLA_2_ reactivity was strong in particular areas of tissue where some macrophages also stained positive (Figure 3E). In ExG samples, the staining intensity of trabecular tissue was lower compared to POAG eyes (Figure 3F), and the strongest staining was in few macrophage-like cells. cPLA_2_ immunoreactivity was barely detectable in trabecular tissue. cPLA_2_ was stained in few macrophages in POAG (Figure 3G) and ExG samples (Figure 3H).

### Photoshop-based image analysis of sPLA_2_-IIA, sPLA_2_-V, iPLA_2_, and cPLA_2_

**Figure 4:**
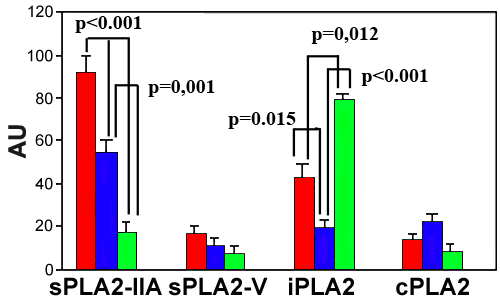
Photoshop-based image analysis of sPLA_2_-IIA, PLA_2_-V, iPLA_2_, and cPLA_2_ levels in TM. Cryostat sections were obtained from POAG patients (red bars), ExG patients (blue bars), or from healthy donor eyes (green bars). The results are mean (arbitrary units)±SEM of six POAG patients, six ExG patients or three healthy donor eyes. Each set of experiments was performed in triplicate.

Because there were semiquantitative differences in PLA_2_s levels between POAG and ExG samples, we analyzed the samples via Photoshop-based image analysis [55]. Cryostat sections obtained from healthy donor eyes served as controls. The level of sPLA_2_-IIA was significantly higher in POAG samples compared to those in ExG (p<0.001) or in control (p<0.001; Figure 4). Expression of sPLA_2_-IIA in ExG was also statistically higher (p=0.001) compared to control group. The immunostaining of sPLA_2_-V was slightly higher in POAG samples compared to ExG patients or control, but there were no significant differences between each experimental group (POAG versus ExG, p=0.997; POAG versus control, p=0.301; ExG versus control patients, p=0.317). Expression of iPLA_2_ was the highest in control group when compared to POAG (p=0.012) or ExG (p<0.001). In POAG, iPLA_2_ level was also significantly higher (p=0.015) compared to ExG. Analysis of cPLA_2_ levels showed no significant differences between each experimental group (POAG versus ExG, p=0.367; POAG versus control, p=0.841; ExG versus control patients, p=0.368).

### Confocal microscopy

**Figure 5:**
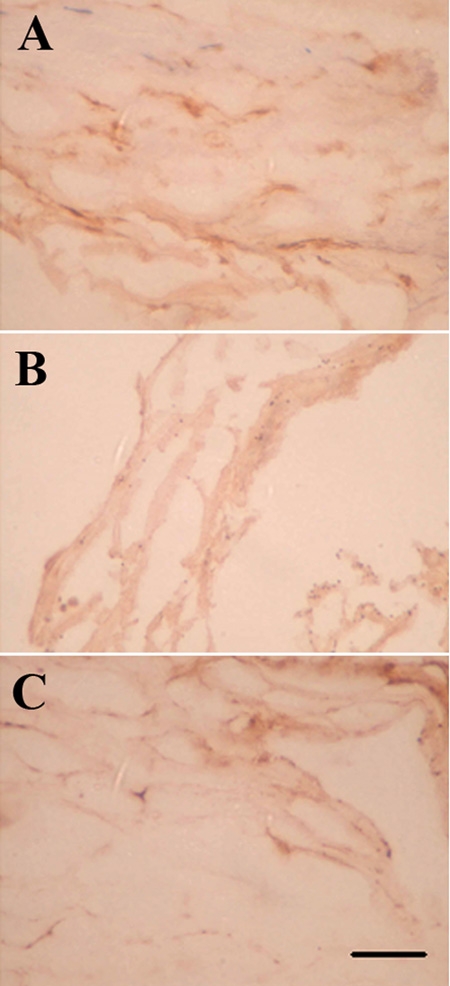
Immunostaining of macrophages. The number of macrophages is increased in POAG (**A**) when compared to ExG (**B**) or control samples (**C**). The scale bar is equal to 50 μm.

**Figure 6:**
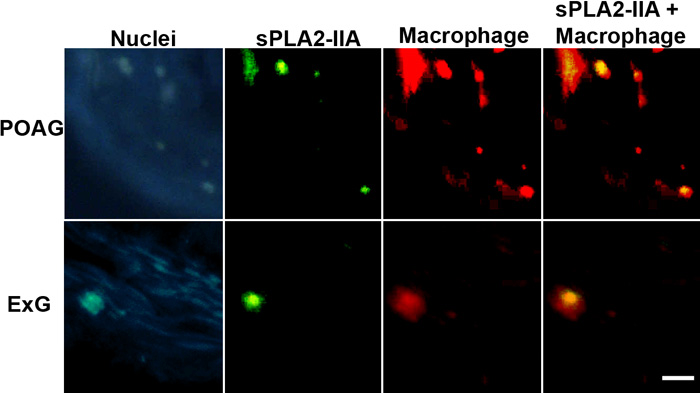
Colocalization of sPLA_2_-IIA and macrophages in POAG and ExG. The first column shows nuclear staining (far red shown in blue) with SYTO 62. The second column shows sPLA_2_-IIA staining (green), and the third column shows macrophages (red). Combined image (sPLA_2_-IIA + macrophage) in the fourth column shows the colocalization of sPLA_2_-IIA and macrophage (yellow). The scale bar is equal to 50 μm.

Immunohistochemical studies showed increased number of macrophages in POAG samples when compared to ExG or control (Figure 5). Furthermore, our results showed clear differences in expression levels of sPLA_2_-IIA and iPLA_2_ in POAG and ExG samples, and immunohistochemical stainings gave indication that both enzymes might also be present in macrophages. Therefore, we next evaluated whether sPLA_2_-IIA could be in macrophages. We carried out confocal microscopy experiments with double antibody staining for sPLA_2_-IIA and macrophages to demonstrate their colocalization and to provide further support for the macrophage derived sPLA_2_-IIA, especially in POAG specimens (Figure 6). Close examination revealed that some of the macrophages showed no colocalization with sPLA_2_-IIA. Interestingly, immunoreactivity of sPLA_2_-IIA positive macrophages was unevenly distributed in POAG samples. In contrast, the number of macrophages was lower, and sPLA_2_-IIA positive macrophages were rarely found in ExG tissue (Figure 6).

### Western blot analysis of PLA_2_s in normal human trabecular meshwork

**Figure 7:**
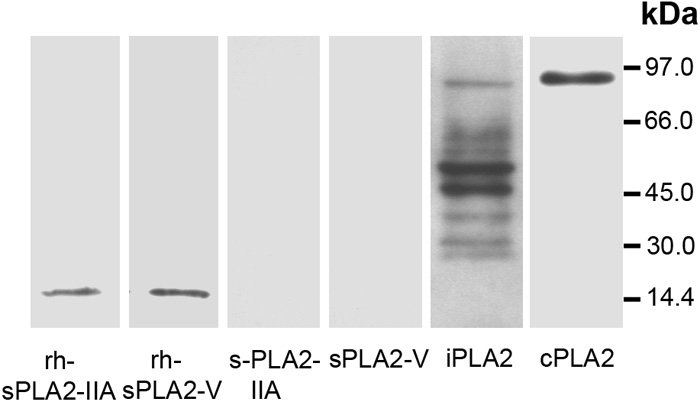
Western blot analysis. Human normal TM samples (10 μg protein) were subjected to SDS-PAGE followed by Western blot analysis using sPLA_2_-IIA, sPLA_2_-V, iPLA_2_, or cPLA_2_ antibody. Recombinant human sPLA_2_-IIA (rh-sPLA_2_-IIA; 200 ng) and sPLA_2_-V (rh-sPLA_2_-V; 200 ng) were used as positive controls. Molecular mass markers are indicated on the right. Results are representative of at least three separate experiments.

No expression of sPLA_2_-IIA or sPLA_2_-V was detected in normal human TM, while about 85 kDa band corresponding to cPLA_2_ was detected (Figure 7). Furthermore, we detected a minor (about 80 kDa) band corresponding to full-length iPLA_2_ and two major (about 55 and 50 kDa) forms. A weak expression of the 40- and 30 kDa forms was also detected. These bands were probably products of alternative splicing or proteolytic degradation.

## Discussion

In the present study, we performed immunohistochemistry to determine the expression of PLA_2_s in chamber angles from normal eyes and in surgical specimens from POAG and ExG patients. To our knowledge, this is the first study to examine this expression. Our results demonstrated significant differences in PLA_2_ levels. We found (1) sPLA_2_-IIA or sPLA_2_-V was not present in normal TM; (2) iPLA_2_ predominated in normal human TM; (3) labeling was strong in the uveal and corneoscleral meshwork; and (4) staining of juxtacanalicular meshwork was only moderate in density. cPLA_2_ was also expressed in normal human TM, and the staining intensity of cPLA_2_ was considerably weaker when compared to iPLA_2_. Furthermore, expression of macrophage-derived sPLA_2_-IIA was highly expressed in POAG patients compared to normal controls, and expression of iPLA_2_ was significantly decreased in ExG (Table 1).

**Table 1:** Changes in PLA_2_ levels and macrophage number in POAG and ExG when compared to normal tissue.

Glaucoma	sPLA_2_IIA expression	sPLA_2_-V expression	iPLA_2_ expression	cPLA_2_ expression	Macrophages
POAG	↑↑	Normal	↓	Normal	↑
ExG	↑	Normal	↓↓	Normal	Normal

Our results showed that sPLA_2_-IIA was significantly increased in glaucomatous tissue compared to normal human TM. sPLA_2_-IIA has an important role in pathological conditions. Accumulation of sPLA_2_-IIA has been demonstrated in human inflammatory diseases such as rheumatoid arthritis, ulcerative colitis, and sepsis [56-58]. The reaction products of sPLA_2_-IIA are lysophospholipids and AA, which are precursors of potent inflammatory mediators such as platelet-activating factor and eicosanoids. Moreover, sPLA_2_-IIA has a high affinity for several proteoglycans such as glypican, decorin, and versican [59]. The biological actions of sPLA_2_-IIA might be governed by interactions with these proteoglycans in the ECM of the TM. We found the expression of sPLA_2_-IIA was significantly higher in POAG samples when compared to ExG or to control. It seems likely that the sPLA_2_-IIA detected in the TM of POAG patients was primarily macrophage derived because it was not present in healthy TM and expression was seen in macrophages present in the TM. It is well established that macrophages have a great secretory capacity for sPLA_2_-IIA at certain stages of activation [60]. Furthermore, in atherosclerosis, macrophage-specific sPLA_2_-IIA has been shown to increase oxidative stress [61]. Thus our results are in concordance with the growing evidence that inflammation and oxidative stress play an important role in the pathogenesis of glaucoma [51,62,63]. In ExG, the lower expression of sPLA_2_-IIA in ExG does not exclude the role of inflammation in the pathogenesis of ExG; it is different because expression and the number of sPLA_2_-IIA positive macrophages are lower when compared to POAG (Table 1).

Histological and morphologic studies have demonstrated that POAG differs from ExG histopathologically. Loss of structural stability and flexibility of the TM, disorganization of the normal juxtacanalicular tissue structure, and increased trabecular pigmentation are typical histopathologic clinical findings in ExG [14,15], whereas POAG is characterized by increased juxtacanalicular plaque and decreased cellularity in the TM [64]. However, mechanisms responsible for these differences in the TM are still unknown. Pathophysiological differences in patients with POAG or ExG was supported further by our finding that expression of iPLA_2_ was significantly lower in ExG samples compared to POAG.

Aqueous humor leaves the eye by passing through intratrabecular spaces in the TM before entering Schlemm's canal [50]. Endothelial cells lining Schlemm's canal and the juxtacanalicular tissue of the TM are expected to be the principal site of outflow resistance [50,65,66]. The physiological functions of trabecular cells are essential for maintaining a normal IOP. It is believed that changes in trabecular ECM, contractility, and cell density may interfere with the normal function of the TM, thereby leading to glaucoma [50,64,67]. It is interesting that iPLA_2_ may have the potential to participate in monocyte chemotaxis, relaxation, contraction, apoptosis, and calcium entry [45,46,48,49,68]. Therefore, iPLA_2_ has many functional characteristics that are important for normal TM cells. Our results showed that iPLA_2_ was expressed in normal human TM, and therefore, we speculate that iPLA_2_ may have a function to maintain normal physiological functions in the TM. The molecular mass of full-length iPLA_2_ is about 80 kDa, and it is present predominantly as 50- and 55 kDa forms, which are most likely ankyrin-iPLA_2_ splice variants [69]. Traditionally, iPLA_2_ has been regarded as a housekeeping enzyme for remodeling and maintenance of membrane phospholipids [42]. Therefore, in ExG eyes, phospholipids remodeling may be dramatically reduced in TM cells. Based on the biological functions proposed for iPLA_2_ it is tempting to speculate that dramatic decrease of the enzyme levels in TM cells may enhance the development of these pathological conditions.

During recent years another sPLA_2_, sPLA_2_-V has been implicated in inflammatory signaling. sPLA_2_-V has been shown to be expressed in a species-dependent manner in mouse cells [70]. In humans, sPLA_2_-V appears to substitute for sPLA_2_-IIA in airway epithelium cells [71]. cPLA_2_ is the only PLA_2_ known to date that is specific for AA at sn-2 position of phospholipids [40]. The activity of cPLA_2_ is important during inflammation because AA is the substrate for the production of prostaglandins and leukotrienes. We show in the present study that normal expression of sPLA_2_-V or cPLA_2_ is low, and there are no significant differences in levels of sPLA_2_-V or cPLA_2_ between healthy, POAG, and ExG tissue. Furthermore, we demonstrated that in normal human TM, cPLA_2_ is a protein with molecular weight about 85 kDa, which is a value typically reported for cPLA_2_ [18].

In summary, we have studied the expression of sPLA_2_-IIA, sPLA_2_-V, iPLA_2_, and cPLA_2_ in the TM of POAG and ExG and compared these levels to healthy controls. The present study provides new information about the expression of PLA_2_s in glaucoma. Distinct levels of sPLA_2_-IIA and iPLA_2_ in POAG and ExG further support the hypothesis that POAG and ExG have different pathogenic mechanisms. During oxidative stress iPLA_2_ recognizes and removes oxidized phospholipids from cell membranes. Due to low expression of iPLA_2_ in ExG the protection against oxidative stress is much worse compared to that in POAG, enhancing the loss of cell function. IOP tends to be greater in ExG than in POAG, and therefore, decreased expression of iPLA_2_ may be a link between increased IOP and loss of structural stability and flexibility of TM cells in ExG. sPLA_2_-IIA has been proposed as an inflammatory marker of cardiovascular disease, and therefore, higher expression of macrophage-derived sPLA_2_-IIA in POAG compared to normal controls supports the view that vascular diseases and POAG may have common pathophysiological mechanisms. Our findings may provide a biochemical basis for the development of new therapeutic agents for POAG and ExG.
